# Effect of meteorological factors on the seasonal prevalence of dengue vectors in upland hilly and lowland Terai regions of Nepal

**DOI:** 10.1186/s13071-019-3304-3

**Published:** 2019-01-18

**Authors:** Reshma Tuladhar, Anjana Singh, Megha Raj Banjara, Ishan Gautam, Meghnath Dhimal, Ajit Varma, Devendra Kumar Choudhary

**Affiliations:** 1 Central Department of Microbiology, Tribhuvan University, Kathmandu, Nepal; 2 Amity Institute of Microbial Technology, Amity University, Noida, UP India; 3 Natural History Museum, Tribhuvan University, Kathmandu, Nepal; 4 Nepal Health Research Council, Ministry of Health and Population, Ramshah Path, Kathmandu, Nepal

**Keywords:** *Aedes aegypti*, *Aedes albopictus*, Vector indices, Temperature, Rainfall, Relative humidity

## Abstract

**Background:**

The expansion of dengue vectors from lowland plains to the upland hilly regions of Nepal suggests the likelihood of increased risk of dengue. Our objective was to assess the effects of meteorological variables on vector indices and populations of dengue vectors in two different ecological regions of Nepal. An entomological survey was conducted in Kathmandu and Lalitpur (upland) and Chitwan (lowland) of Nepal in three different seasons from July 2015 to May 2016. The effect of meteorological variables on vector indices (house index, container index and Breteau index) and *Aedes* spp. population abundance was analyzed. A gamma regression was used to fit the models for vector indices and a negative binomial regression was used to model *Aedes* spp. population abundance.

**Results:**

Monsoon season showed higher values for vector indices and vector populations compared to post-monsoon and pre-monsoon. Overall, the factor temperature-rainfall effect had a more significant influence on vector indices compared to relative humidity. The regression models showed that relative humidity has a greater impact in Chitwan than in Kathmandu. Variation was observed in the effect of predictor variables on *Aedes aegypti* and *Ae. albopictus* abundance.

**Conclusions:**

Temperature and rainfall contribute to the vector indices in the upland hilly region while relative humidity contributes in the lowland plains. Since vector prevalence is not only linked to meteorological factors, other factors such as water storage practices, waste disposal, sanitary conditions and vector control strategy should also be considered. We recommend strengthening and scaling up dengue vector surveillance and control programmes for monsoon season in both upland and lowland regions in Nepal.

**Electronic supplementary material:**

The online version of this article (10.1186/s13071-019-3304-3) contains supplementary material, which is available to authorized users.

## Background

Dengue is an emerging health problem in Nepal which is caused by dengue virus (DENV) and is transmitted to humans mainly by *Aedes aegypti* and *Ae*. *albopictus* mosquitoes. An estimated half of the world population is at risk of dengue with tropical and sub-tropical regions being most vulnerable [1]. It has been calculated that 390 million dengue infections occur per year, of which 96 million manifest as severe [2]. Dengue has become a major public health concern due to its rapid expansion with epidemics being confirmed in 128 countries, including those in Southeast Asia, the Americas, Africa and the western Pacific and Mediterranean regions [3, 4].

Dengue was first reported in Nepal in 2004 from Chitwan District, while the first outbreak occurred in 2006 with confirmed cases from nine districts of the lowland region, also known as Terai in Nepal [5, 6]. Following the 2006 outbreak, dengue cases were reported every year. The next major outbreak occurred in 2010 with 917 cases including five deaths that affected 12 districts of central and western Nepal [7]. Subsequent large outbreaks in the years 2013 and 2016 resulted in 683 cases from 15 districts and 1527 cases from 30 districts, respectively [8]. The periodic outbreaks together with the expansion of dengue affected districts suggest a perpetual threat of dengue in the country.

Previously, dengue cases were reported only from the warmer lowland regions of Nepal. However, the first case of dengue in a patient from Kathmandu (upland region) without a recent history of travel to a dengue-affected area in 2010 [9] was an indication that the upland hilly region of Nepal is also vulnerable to dengue. This warning was supported by a report of finding of the primary vector *Ae. aegypti* for the first time in Kathmandu in 2009 [10] and by later studies in 2014 [11, 12]. Earlier entomological investigation had reported the presence of secondary vector *Ae. albopictus* in Nepal and ascertained that *Ae. aegypti* did not exist here [13]. However, stable populations of both vectors have already been established from the lowlands to the middle mountain region at an altitude of 1310 m [14]. Thus, expansion of the geographical range of dengue can be attributed to the spread of *Ae. aegypti* [15].

Assumptions have been made that the increase in incidence of vector-borne disease like dengue is linked to the climate change as a consequence of global warming [16–18]. Temperature, rainfall and humidity are considered important factors for the survival and development of vectors and the transmission rates of vector-borne pathogens [19, 20]. While warmer temperatures facilitate the development of mosquito and viral replication [21], adequate rainfall and humidity affect the breeding habitats and survival of vectors, resulting in increased vector populations [22].

The magnitude and geographical distribution of vector populations have been made based on the calculation of vector indices and container habitats [23]. Many studies have focused on larval indices to predict the risk and transmission of dengue [24–26]. The vector indices considered in this research were house index (HI), container index (CI) and Breteau index (BI). We studied larval indices rather than adult population due to collection of adults being both challenging and labor-intensive. Furthermore, dispersal and translocation of adult populations by various means of transportation may provide inaccurate data.

There are a few published studies on seasonal abundance of *Aedes* vectors in Nepal [12, 14]; however, the statistical relationships between vector indices and meteorological variables have not been studied. The relationship between meteorological variables and mosquito abundance can provide important information in determining the disease risk. Therefore, this study was conducted to investigate population dynamics of *Aedes* vectors, their species composition, and their association with meteorological variables in two different ecological regions of Nepal.

## Methods

### Study area

**Fig. 1:**
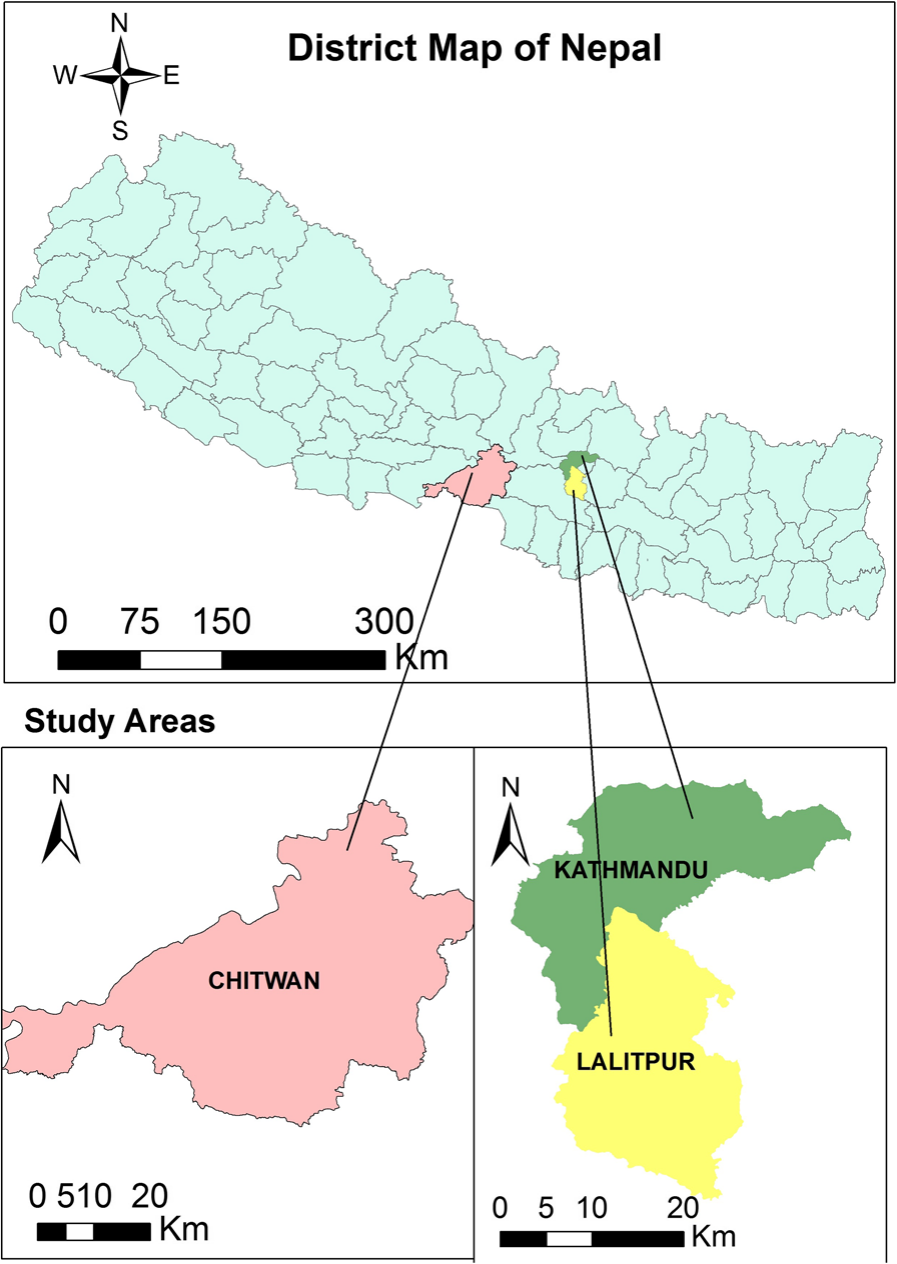
Study areas

Two ecologically different areas of Nepal were selected for this study (Fig. 1). Kathmandu Valley represents an upland hilly region and Chitwan District represents a lowland Terai region of the country. Situated in the central Nepal, Kathmandu Valley has an approximate elevation of 1310 m above mean sea level (masl) and is characterized by a warm temperate climate. The two districts selected from this valley for sample collections were Kathmandu and Lalitpur municipalities. Chitwan District, situated in the subtropical inner Terai belt of Nepal with an elevation of 415 masl represents lowland.

### Data collection

An entomological survey was carried out in three seasons from July 2015 to May 2016 to determine the variation in the abundance of the vector populations in different seasons. The period from July to September was considered monsoon, October to December as post-monsoon and March to May as pre-monsoon [27]. Monsoon and pre-monsoon are warmer seasons compared to post-monsoon (Table 1).

**Table 1:** Average meteorological records in Kathmandu Valley and Chitwan District of Nepal

Location	Season	Minimum temperature (°C)^a^	Maximum temperature (°C)^a^	Rainfall (mm)^a^	Relative humidity (%)^a^
Kathmandu Valley (hilly region)	Monsoon	19.6 ± 0.62	29.33 ± 0.21	370.7 ± 157.20	82.3 ± 1.50
	Post-monsoon	9.2 ± 4.70	23.2 ± 4.20	22.5 ± 39.00	79.6 ± 2.50
	Pre-monsoon	13.03 ± 3.30	27.23 ± 3.00	106.4 ± 59.60	66.8 ± 3.40
Chitwan District (plain land)	Monsoon	25.13 ± 0.47	33.9 ± 0.52	339.2 ± 174.75	84.82 ± 0.45
	Post-monsoon	14.1 ± 5.25	27.6 ± 4.41	1.4 ± 2.42	88.5 ± 4.12
	Pre-monsoon	18.3 ± 3.76	32.7 ± 2.81	56.7 ± 50.77	75.1 ± 3.70

^a^Data are average of monthly data of each season ± standard deviation (SD)

We selected 10 locations each from Kathmandu and Chitwan districts, and 9 locations from Lalitpur District for the vector survey. From each district, 100 house premises were inspected for *Aedes* larvae from potential water-holding containers around the house (outdoor). Types of containers were recorded and the container preferences of *Aedes* vector breeding were assessed by calculating the breeding preferences ratio (BPR) which is defined as the ratio of number of containers infested (positive) with larvae to the number of water-holding containers examined [28]. The water-holding containers with immature *Aedes* mosquitoes (larvae) were considered as positive containers. The vector indices considered in the survey were: house index (HI) (percentage of houses with containers positive for *Aedes* larvae); container index (CI) (percentage of water-holding containers infested with larvae); and Breteau index (BI) (number of positive containers per 100 houses inspected) [23]. These indices were considered in order to assess the prevalence of *Aedes* vectors.

Daily records of rainfall (mm), relative humidity (%), maximum temperature (°C) and minimum temperature (°C) from the weather station located < 10 km from house inspected in the lowland and < 5 km from house inspected in upland (hills) were obtained from the Department of Hydrology and Meteorology, Kathmandu.

### Statistical analysis

Vector indices (HI, CI and BI) and *Aedes* spp. numbers were response variables. The factor analysis with principal components analysis (PCA) method was used to reduce the number of variables or to remove the effect of multicollinearity of meteorological variables. TempRain and RelHumidity were two components derived from factor analysis. The predictor variables considered were Kathmandu, Chitwan, monsoon, post-monsoon, TempRain, RelHumidity, K_TempRain, K_RelHumidity, C_TempRain and C_RelHumidity. K and C were dummy variables which indicated Kathmandu and Chitwan, respectively, with reference to Lalitpur. Similarly, monsoon and post-monsoon were dummy variables which signified monsoon and post-monsoon, respectively, with reference to pre-monsoon.

Data collection on response variables was cross-sectional in nature although different seasons were considered. Data were based on the sample from households in three different districts. Each household was surveyed enumerated three times to note the response of vector in three different seasons. Data on meteorological predictor variables were also recorded three times. As the response variables, HI, CI and BI, are continuous in nature, regression models for count response variables such as a Poisson regression or negative binomial models were not suitable. The repeated sample size was too small to run a generalized linear mixed model (GLMM). The inclusion of interaction terms also prevented its further use since models with interaction terms need a sufficient sample size. The final dataset had only 87 sample observations. Thus, we used generalized linear model (GLM) since it can fit even when the response variables are non-linear in nature and have outliers. The response variables, *Ae. aegypti* and *Ae. albopictus* numbers, have a count outcome, hence a Poisson or negative binomial model was possible. Since the standard deviation was greater than mean, we used a negative binomial regression for vector abundance.

Some models were fitted with a log-link function in GLM. For this, the response variable must be positive, but HI, BI and CI have some zero values. To avoid this complication, a constant quantity of 1 was added to each of these response variables. The addition of a constant to a variable did not affect the mean and variance of that variable. These variables were then named as HI_1, BI_1 and CI_1. Generalized models were fitted to the latter response variables separately on a given set of meteorological predictor variables along with some dummy variables. Moreover, the models included some interaction terms based on the fitting criteria. The best-fit models were selected and interpreted.

All statistical analyses were conducted using SPSS Statistics software v.21. GraphPad Prism v.7 was used to construct scatter plots and bar diagrams.

## Results

**Fig. 2:**
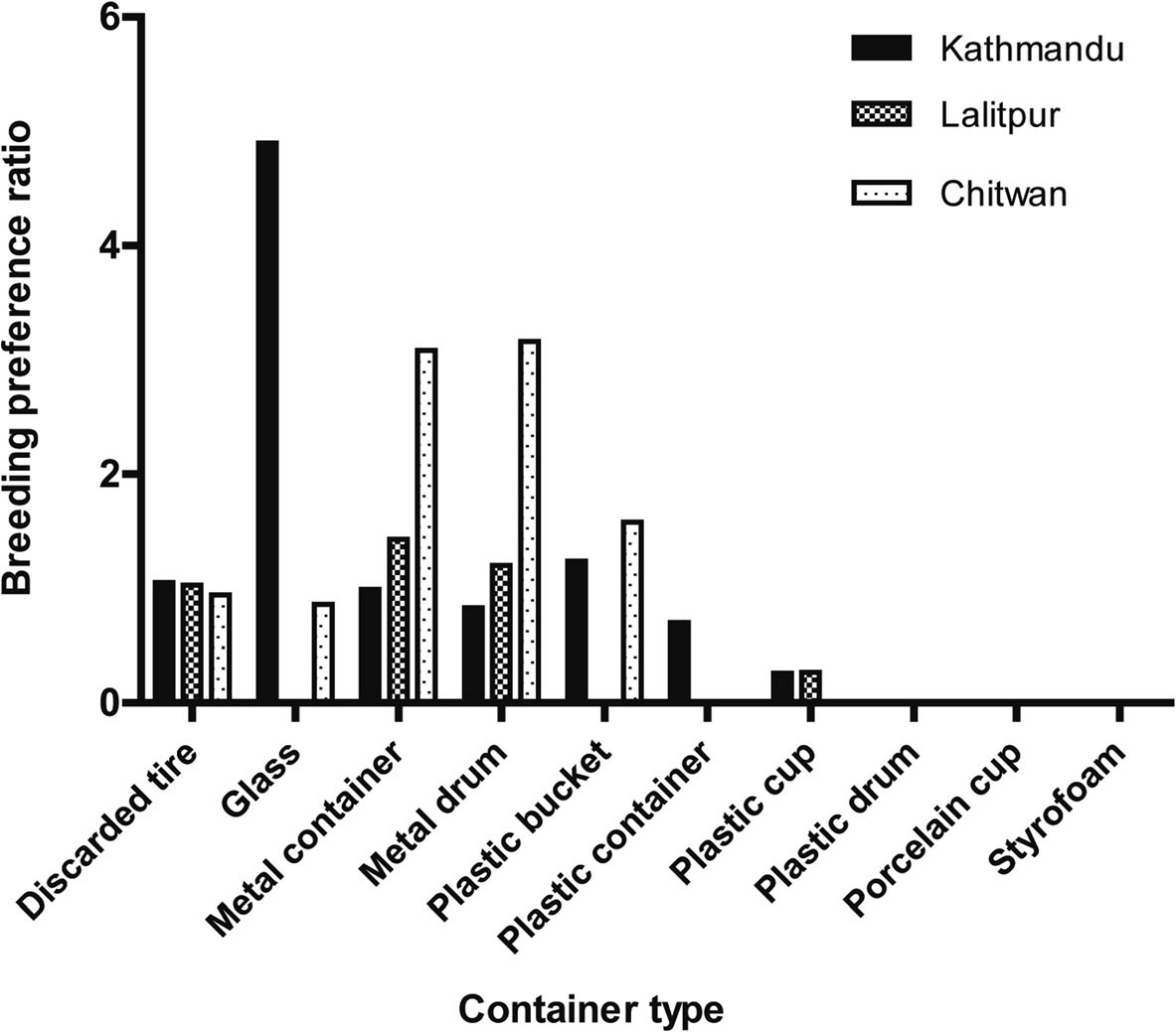
Breeding preference ratio (BPR) of different containers positive for *Aedes* vector breeding

A total of 300 houses (100 houses in each location) were surveyed, out of which 64 houses were found to be positive for *Aedes* breeding containers. Within the premises of houses surveyed, 782 water-holding containers (*n* = 332 in Kathmandu; *n* = 277 in Lalitpur; and *n* = 173 in Chitwan) were identified and *Aedes* larvae was present in 220 water-holding containers (*n* = 67 in Kathmandu; *n* = 100 in Lalitpur; and *n* = 53 in Chitwan). Containers found in each house were recorded to determine the dominant mosquito breeding containers in different locations. Since the survey was conducted in an urban area, breeding habitats found were primarily artificial containers. In terms of number, discarded tire was the dominant breeding container in all three locations. The highest number of discarded tires (*n* = 92) with a presence of *Aedes* larvae was found in Lalitpur, while the least number (*n* = 46) was found in Chitwan. Containers made of materials like metal and plastic were common breeding habitats (Additional file 1: Table S1). The BPR was observed to be the highest for glass containers (4.9) in Kathmandu, metal containers (1.43) in Lalitpur and metal drum containers (3.16) in Chitwan (Fig. 2).

The vector indices HI, CI and BI were found to be the highest during monsoon season followed by post-monsoon and the lowest during the pre-monsoon season (Table 2). The highest vector indices were recorded in Lalitpur during monsoon season (HI: 21.2; CI: 41.8; BI: 0.1) followed by Chitwan (HI: 20.8; CI: 36; BI: 0.05) and Kathmandu (HI: 19.7; CI: 24.3; BI: 0.06). The vector indices values were observed to be the lowest in Chitwan during post-monsoon season (Table 2). Since no water-holding containers were identified in the premises of the houses surveyed in Chitwan during the pre-monsoon season, vector indices were not assessed in this area for this period. HI values were > 5% during monsoon season and varied between 0.1–5% during post-monsoon season.

**Table 2:** Entomological indices (HI, CI and BI) in study locations at different seasons

Location	*n*	Larval indices in monsoon			Larval indices in post-monsoon			Larval indices in pre-monsoon		
		HI	CI	BI	HI	CI	BI	HI	CI	BI
Kathmandu	10	19.7	24.3	0.06	3.02	15.20	0.0060	0.59	4.0	0.0020
Lalitpur	9	21.2	41.8	0.10	3.00	12.06	0.0067	3.17	7.4	0.0067
Chitwan	10	20.8	36.0	0.05	2.70	11.10	0.0030	–	–	–

*Abbreviations*: *BI* Breteau index, *CI* container index, *HI* house index

**Fig. 3:**
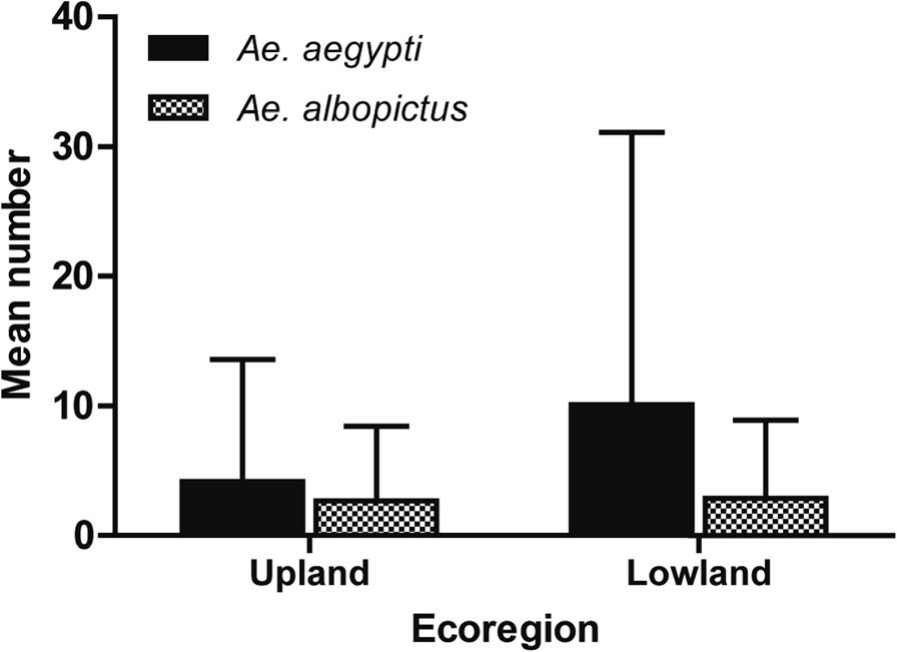
Comparision of *Ae. aegypti* and *Ae. albopictus* number in upland hilly region and lowland Terai region

The highest numbers of *Aedes* larvae were detected during monsoon season (Additional file 2: Figure S1). The average number of *Ae. aegypti* larvae was higher in the lowland Terai region compared to upland hills where the average number of *Ae. albopictus* was almost identical in both regions, indicating *Ae. aegypti* to be the dominant species in lowland (Fig. 3).

**Fig. 4:**
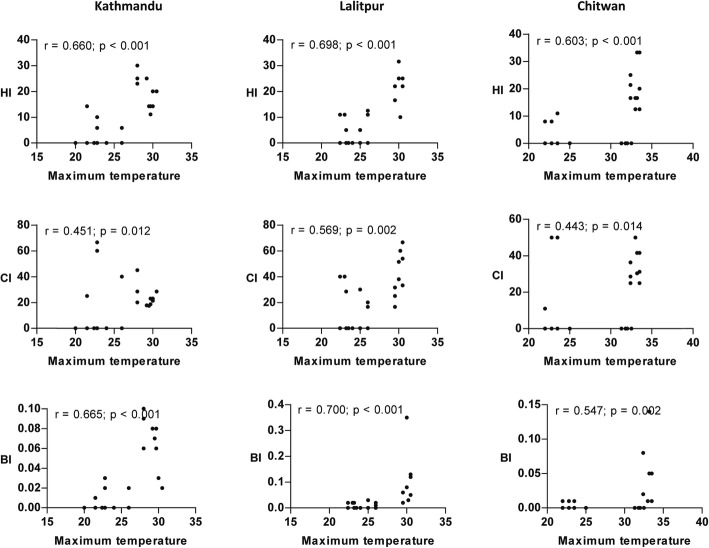
Scatterplot of the relationship between maximum temperature (°C) and vector indices (HI, CI and BI) in three different locations

**Fig. 5:**
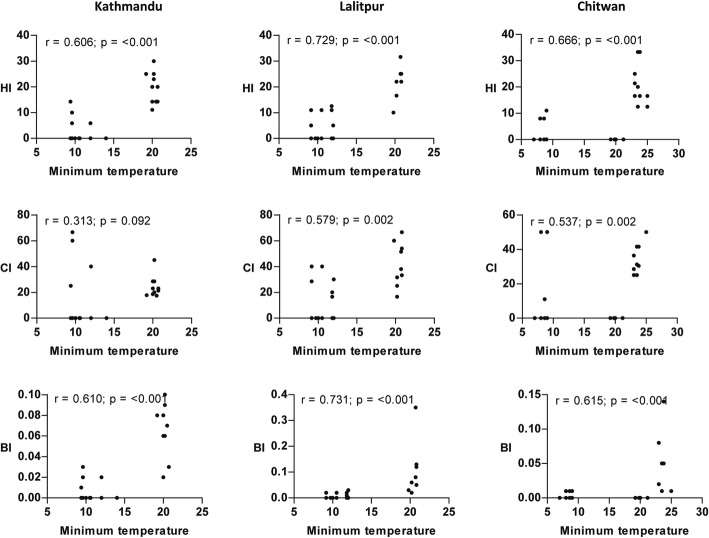
Scatterplot of the relationship between minimum temperature (°C) and vector indices (HI, CI and BI) in three different locations

**Fig. 6:**
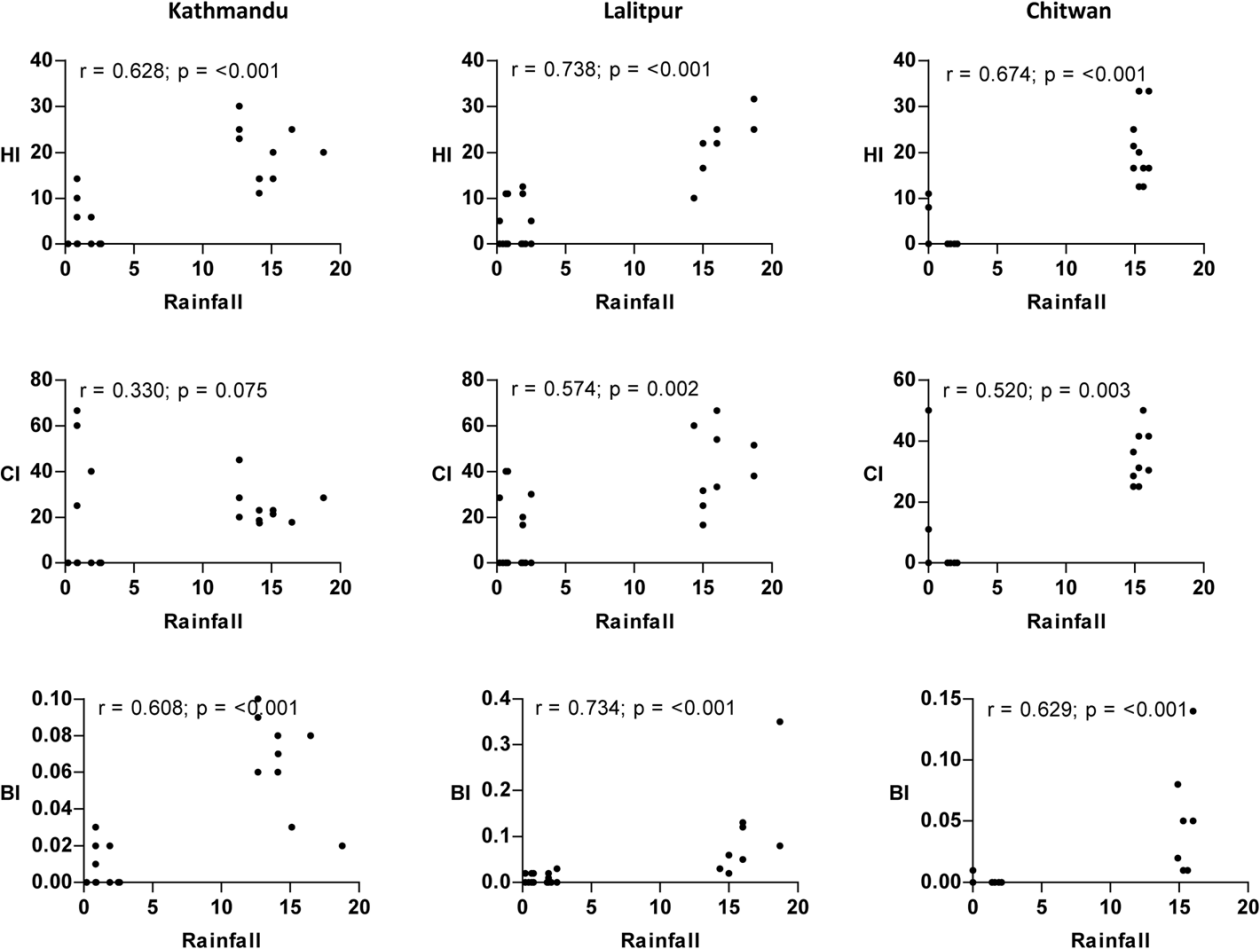
Scatterplot of the relationship between rainfall (mm) and vector indices (HI, CI and BI) in three different locations

**Fig. 7:**
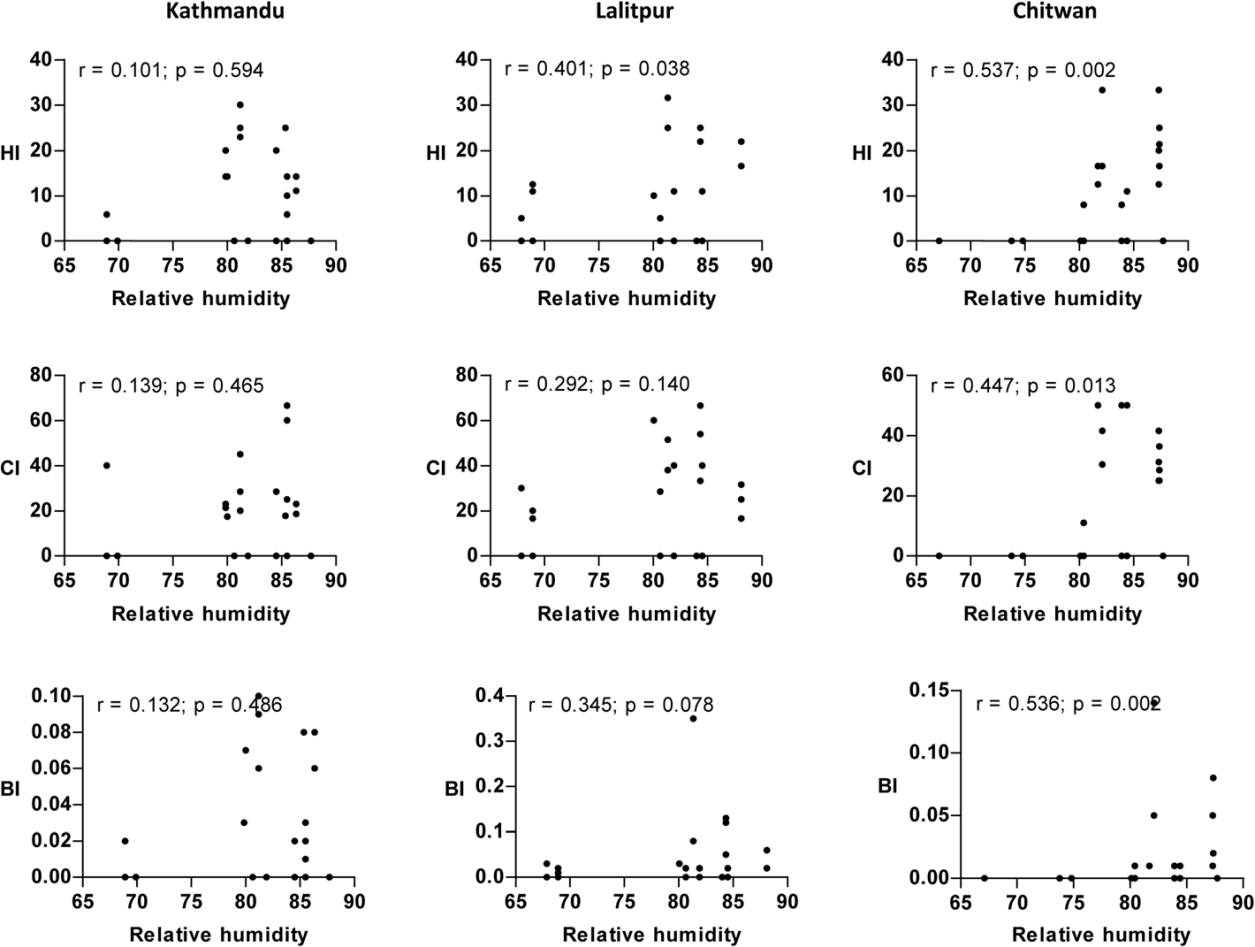
Scatterplot of the relationship between relative humidity (%) and vector indices (HI, CI and BI) in three different locations

**Fig. 8:**
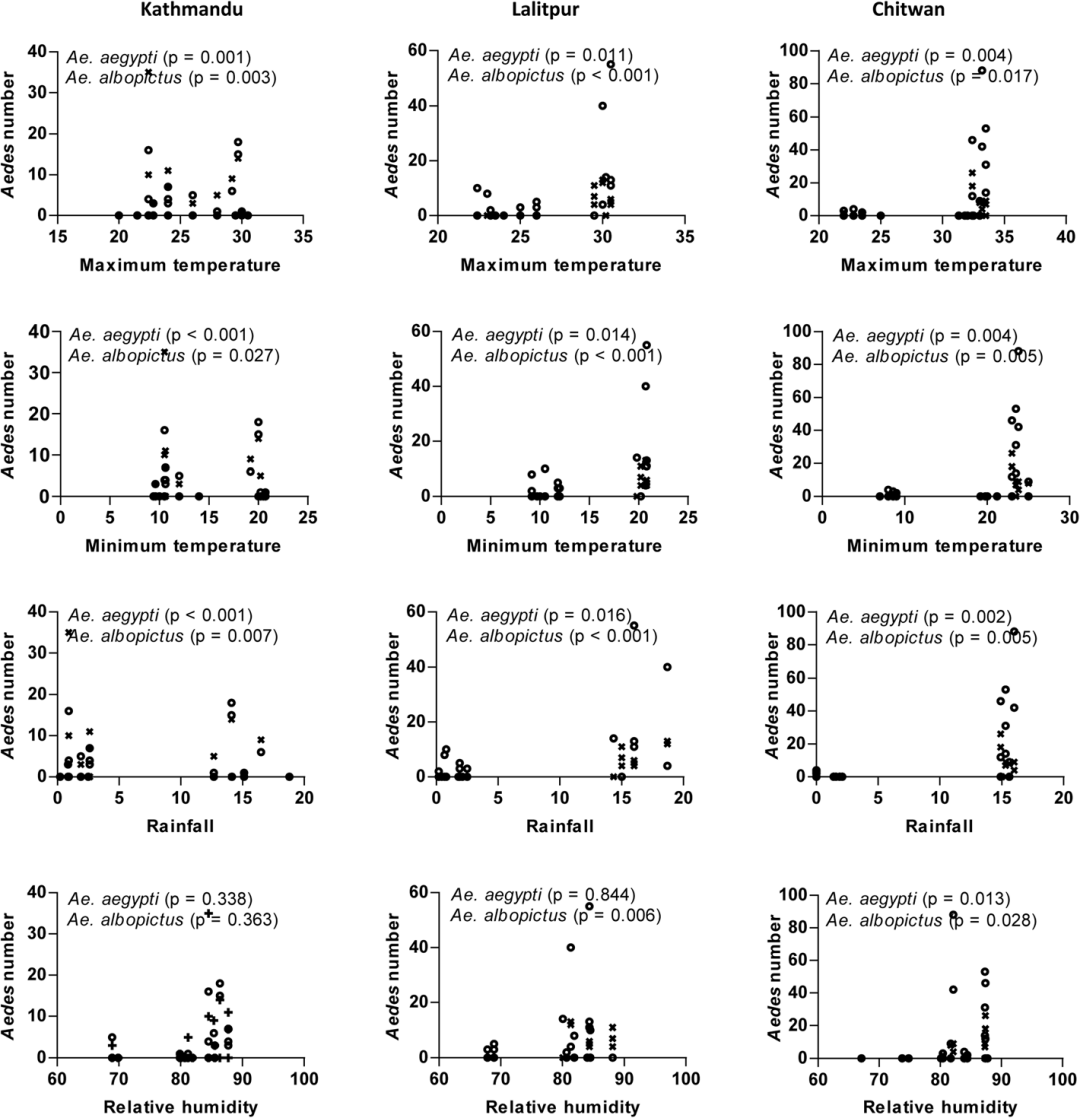
Relationship of *Ae. aegypti* (circles) and *Ae. albopictus* (crosses) abundance with meteorological variables in three different locations

A significant positive relationship (*P* < 0.05) between maximum temperature and vector indices was observed from the scatterplot (Fig. 4). Similarly, the correlation of minimum temperature and rainfall with vector indices was nearly identical to the results of maximum temperature in all locations except for CI in Kathmandu Valley where a poor correlation was observed (Figs. 5 and 6). The strength of the relation of HI and BI with the meteorological variables was comparatively better than CI. Relative humidity showed a significant weak positive correlation with vector indices in Chitwan, but no linear correlation in the hilly regions (Fig. 7). Despite the significant correlation of temperature and rainfall with vector abundance in both ecological regions, relative humidity showed a differential relationship (Fig. 8).

Some important predictor variables were omitted due to their high multicollinearity effect in the regression models. To keep these variables in the model, a PCA method was adopted. This method created two new predictor variables termed as factors or principal components. Factor 1 was designated as temperature-rainfall effect (TempRain) and factor 2 as relative humidity effect (RelHumidity) (Additional file 3: Table S2). The newly generated variables were not correlated.

Generalized linear models (GLM) were fitted for response variables on given predictor variables along with some dummy variables based on the fitting criteria. Both a deviance test and omnibus test supported that six models Normal (HI_1) with identity link function, Normal (HI_1) with log link function, Gamma regression (HI_1) with identity link function, Gamma (HI_1) with log link function, Tweedle_1.5 (HI_1) with identity link function and Tweedle_1.5 (HI_1) with log link function were well fitted to the data (Additional file 4: Table S3). In order to select best-fit model within the six types of GLM of HI_1, the criteria of lowest value of AIC (or BIC), mean deviance (-2LL/df) close to one, and dispersion parameter less than one, were used. Gamma (HI_1) with identity link function and Gamma (HI_1) with log link function were preferred as the best-fit models. Gamma (HI_1) with log link function seemed better than Gamma (HI_1) with identity link function since the former model included five significant predictor variables compared to the latter model. Hence, Gamma (HI_1) with log link function was further modified such that it contained all the significant predictor variables. Several possible subsets of this model were fitted with the significant predictors. The final model was selected and is listed in Additional file 4: Table S3 (models 7, 8 and 9) and Table 3. Three gamma regression models for HI_1 were selected as they satisfied all the criteria of model fit (Additional file 4: Table S3). Furthermore, they included only significant predictor variables and the coefficients of each of these models were interpreted.

**Table 3:** Gamma regression models of HI_1

Parameter	Model 4		Model 7		Model 8		Model 9	
	b (SE)	*P* ^a^	b (SE)	*P* ^a^	b (SE)	*P* ^a^	b (SE)	*P* ^a^
(Intercept)	0.579 (0.384)	0.132	0.329 (0.251)	0.190	1.995 (0.116)	<0.0001	1.825 (0.099)	<0.0001
Chitwan	-0.470 (0.326)	0.150			-0.637 (0.198)	0.001		
Kathmandu	-0.279 (0.223)	0.211						
Monsoon	2.818 (0.996)	0.005	2.90 (0.374)	<0.0001				
Post-monsoon	1.201 (0.559)	0.032	1.148 (0.361)	0.001				
TempRain	0.143 (0.67)	0.831			0.734 (0.094)	<0.0001	0.900 (0.121)	<0.0001
RelHumidity	-0.540 (0.211)	0.010	-0.542 (0.192)	0.005	0.340 (0.116)	0.003		
C_TempRain	-0.096 (0.269)	0.721					-0.429 (0.202)	0.034
C_RelHumidity	0.619 (0.304)	0.042	0.603 (0.213)	0.005	0.749 (0.207)	<0.0001	1.041 (0.203)	<0.0001
K_TempRain	-0.158 (0.257)	0.538						
K_RelHumidity	0.391 (0.208)	0.060	0.394 (0.196)	0.044			0.569 (0.108)	0.001

*Abbreviation*: *SE* standard error

^a^Wald Chi-square test

Model 4 included all 10 predictor variables. However, the fitting of the models showed only five of them as highly significant predictors under the Wald Chi-square test (*P* < 0.05) (Table 3). Hence, it did not meet the requirement of only significant predictors from the fitting of gamma regression of HI_1 with log link function. We interpreted the coefficients of the predictors, dummy variables Chitwan and Kathmandu (as location variables) have no influence on HI_1. Similarly, monsoon and post-monsoon (as seasonal variables) have significant consequence. TempRain as main effect, C_TempRain and K_TempRain as interaction effects have insignificant influence on HI_1. RelHumidity, C_ RelHumidity and K_ RelHumidity as main and interaction effects have significant consequence on HI_1.

The partial regression coefficients are expressed in exponential form, i.e. exp (b_i_), so that interpretation is simple and straightforward. Exponential value >1 indicated greater effect of the predictor on the response variable and exponential value < 1 indicated less effect of the predictor on the response variable. Exponential of the zero value of the coefficient is one. When interpreting partial regression coefficients, a negative sign indicates a decrease in the response variable and a positive sign indicates an increase in the response variable per unit increase in a given predictor keeping the remaining predictors constant.

Chitwan [exp (-0.470) = 0.625] and Kathmandu [exp (-0.279) = 0.756] have values < 1. This means that Lalitpur could have a greater value of HI_1 on average compared to Chitwan and Kathmandu. Moreover, RelHumidity [exp (-0.540) = 0.583], C_TempRain [exp (-0.096) = 0.908] and K_TempRain [exp (-0.158) = 0.854] showed significantly lower effect on HI_1 keeping all other predictors constant. TempRain [exp (0.143) = 1.15], C_RelHumidity [exp (0.619) = 1.857] and K_RelHumidity [exp (0.391) = 1.478] showed a greater impact on HI_1 keeping all other predictors constant. Monsoon [exp (2.818) = 16.74] and post-monsoon [exp (1.201) = 3.32] showed a significant effect on HI_1. Monsoon showed a greater impact on HI_1 compared to post-monsoon and pre-monsoon.

Models 7 and 8 used the subsets of the 10 predictor variables. So, the inclusion of some predictors may result in a variation of the sign and value of the coefficients of the predictors due to the capacity of influence of the predictors on the response variable in a model. Model 7 included five significant predictors: monsoon, post-monsoon, RelHumidity, C_ RelHumidity and K_ RelHumidity. This model excluded TempRain or its interaction terms with location. In the partial coefficients, monsoon demonstrated a value of 2.90 or [exp (2.90) = 18.17] and post-monsoon 1.148 or [exp (1.148) = 3.15]. These two seasons seemed to have a different impact on HI_1. Relatively, HI_1 is likely to increase in the monsoon rather than in the post-monsoon season. The partial regression coefficients of RelHumidity, C_RelHumidity and K_RelHumidity were -0.542 or [exp (-0.542) = 0.582], 0.603 or [exp (0.603) = 1.828] and 0.394 or [exp (0.394) = 1.483], respectively. The effect of relative humidity on HI_1 was greater in Chitwan than in Kathmandu with reference to effect in Lalitpur.

Model 8 included four significant predictors, Chitwan, TempRain, RelHumidity and C_RelHumidity, but excluded season. The partial regression coefficient for Chitwan was -0.637 or [exp (-0.637) = 0.529]. Chitwan showed significantly lower effect on HI_1 with reference to Lalitpur on average. TempRain showed a partial regression coefficient of 0.734 or [exp (0.734) = 2.083], followed by RelHumidity of 0.340 or [exp (0.340) = 1.40], C_RelHumidity of 0.749 or [exp (0.749) = 2.115]. The effect of relative humidity on HI_1 was greater in Chitwan with reference to effect in Lalitpur. The effect was followed by TempRain on HI_1 on average keeping the effect of other predictors constant. Similarly, model 9 also included four significant predictors, TempRain, C_TempRain, RelHumidity, C_RelHumidity and K_RelHumidity, with season excluded. The partial regression coefficient for TempRain was 0.900 or [exp (0.900) = 2.46], followed by C_TempRain of -0.429 or [exp (-0.429) = 0.651], C_RelHumidity of 1.041 or [exp (1.041) = 2.832] and K_RelHumidity of 0.569 or [exp (0.569) = 1.766]. The effect of relative humidity on HI_1 was greater in Chitwan again. TempRain had relatively lower effect on HI_1 in Chitwan compared to relative humidity at the same location with reference to Lalitpur on average after keeping the effect of other predictors constant.

For GLM regression of CI_1, model 12 was selected based on the lowest AIC value among the first five models (Additional file 4: Table S4). Following the modification of Gamma with log link function, model 15 was selected as final model (Table 4). However, this model showed a slightly higher dispersion parameter (i.e. > 1). It was selected for including only significant predictor variables.

**Table 4:** Gamma regression models of CI_1

Parameter	Model 12		Model 15	
	b (SE)	*P* ^a^	b (SE)	*P* ^a^
(Intercept)	1.068 (0.598)	0.074	0.904 (0.358)	0.012
Chitwan	-0.932 (0.486)	0.055	-0.618 (0.257)	0.016
Kathmandu	-0.151 (0.319)	0.637		
Monsoon	2.388 (1.552)	0.124	3.091 (0.519)	<0.0001
Post-monsoon	2.755 (0.776)	<0.0001	2.817 (0.523)	<0.0001
TempRain	0.868 (1.012)	0.391		
RelHumidity	-0.838 (0.303)	0.006	-0.658 (0.229)	0.004
C_TempRain	-0.317 (0.385)	0.409		
C_RelHumidity	1.173 (0.451)	0.009	0.823 (0.263)	0.002
K_TempRain	-0.563 (0.376)	0.134		
K_RelHumidity	0.227 (0.163)	0.441		

*Abbreviation*: *SE* standard error

^a^Wald Chi-square test

Only four predictor variables out of 10 were highly significant predictors in model 12 under the Wald Chi-square test (*P* < 0.05). Thus, it did not meet the requirement of only significant predictors from fitting of gamma regression of CI_1 with log link function. In an attempt to interpret the coefficients of the predictors, dummy variables Chitwan and post-monsoon were significant but Kathmandu and monsoon were not significant. TempRain as a main effect and C_TempRain, K_TempRain and K_ RelHumidity as interaction effects were not significant. However, RelHumidity and C_ RelHumidity were significant as main and interaction effects on CI_1 (Table 4). The coefficients when interpreted in exponential form, i.e. [exp (b_i_)], both had a value < 1 with reference to Lalitpur: Chitwan [exp (-0.932) = 0.394] and Kathmandu [exp (-0.151) = 0.859]. This means CI_1 had more influence in Lalitpur on average. Moreover, RelHumidity [exp (-0.838) = 0.433], C_TempRain [exp (-0.317) = 0.728] and K_TempRain [exp (-0.563) = 0.569] showed significantly lower effect on CI_1 while keeping all other predictors constant. TempRain [exp (0.868) = 2.38], C_RelHumidity [exp (1.173) = 3.23] and K_RelHumidity [exp (0.227) = 1.25] showed greater impact on CI_1 while keeping all other predictors constant. Monsoon [exp (2.388) = 10.89] and post-monsoon [exp (2.755) = 15.72] showed significantly greater effect on CI_1. Post-monsoon showed a greater impact on CI_1 compared to monsoon with reference to pre-monsoon while keeping all other predictors constant.

Model 15 included five significant predictors: Chitwan, monsoon, post-monsoon, RelHumidity and C_RelHumidity. The partial regression coefficients of Chitwan, monsoon, post-monsoon, RelHumidity and C_RelHumidity were -0.618 or [exp (-0.618) = 0.539], 3.091 or [exp (3.091) = 21.99], 2.817 or [exp (2.817) = 16.73] and -0.658 or [exp (-0.658) = 0.52] and 0.823 or [exp (0.823) =2.27], respectively. The effect of monsoon was greatest followed by post-monsoon on CI_1 in average. C_RelHumidity had relatively more effect on CI_1 in Chitwan with reference to Lalitpur.

Similarly, for BI_1 in model 19, Gamma with log link function was selected as it possessed the lowest AIC value (-327.90) among the first six models (Additional file 4: Table S5). Gamma regression with log link function was modified as earlier. The final model selected was model 22 (Additional file 4: Table S5 and Table 5). Although model 19 included all 10 predictor variables, the fitting of the models showed only four predictors as highly significant under the Wald Chi-square test (*P* < 0.05). Thus, it did not meet the requirement of only significant predictors from the fitting of Gamma regression of BI_1 with log link function. Regarding the coefficients of the predictors, effect of Chitwan was significant but Kathmandu, monsoon and post-monsoon were not significant. TempRain as main effect, C_TempRain and K_TempRain as interaction effects have significant consequence, but RelHumidity, C_ RelHumidity and K_ RelHumidity as main and interaction effects were not significant on BI_1. The coefficients were interpreted in the exponential form, i.e. [exp (b_i_)] as earlier. Chitwan [exp (0.028) = 1.028] had a value > 1 but Kathmandu [exp (-0.014) = 0.986] had a value < 1, indicating that BI_1 had more influence in Chitwan than in Kathmandu with reference to Lalitpur on average. RelHumidity [exp (-0.005) = 0.995], C_TempRain [exp (-0.029) = 0.908] and K_TempRain [exp (-0.023)=0.977] all showed significantly lower effect on BI_1 keeping all other predictors constant. TempRain [exp (0.042) = 1.04], C_RelHumidity [exp (0.006) = 1.006] and K_RelHumidity [exp (0.005) = 1.005] showed greater impact on the BI_1 keeping predictors constant. Similarly, monsoon [exp (0.036) = 1.037] and post-monsoon [exp (0.025) = 1.025] showed significant effect on BI_1. Monsoon showed a greater impact on BI_1 compared to post-monsoon with reference to pre-monsoon keeping all other predictors constant.

**Table 5:** Gamma regression models of BI_1

Parameter	Model 19		Model 22	
	b (SE)	*P* ^a^	b (SE)	*P* ^a^
(Intercept)	0.210 (0.0145)	0.154	0.021 (0.0083)	0.011
Chitwan	-0.028 (0.0128)	0.026	-0.021 (0.0144)	0.019
Kathmandu	-0.014 (0087)	0.109	-0.016 (0.0084)	0.051
Monsoon	0.036 (0.365)	0.323	0.054 (0.0144)	<0.0001
Post-monsoon	0.025 (0.0202)	0.214		
TempRain	0.042 (0.0245)	0.089	0.021 (0.010)	0.035
RelHumidity	-0.005 (0.0081)	0.554		
C_TempRain	-0.029 (0.0102)	0.004	-0.024 (0.0089)	0.007
C_RelHumidity	0.006 (0.0119)	0.615		
K_TempRain	-0.023 (0.0098)	0.017	-0.022 (0.0094)	0.021
K_RelHumidity	0.005 (0.0087)	0.593		

*Abbreviation*: *SE* standard error

^a^Wald Chi-square test

Six significant predictors included in model 22 were Chitwan, Kathmandu, monsoon, TempRain, C_TempRain and K_TempRain. Partial regression coefficients of Chitwan, Kathmandu, monsoon, TempRain, C_TempRain and K_TempRain were -0.021 or [exp (-0.021) = 0.979], -0.016 or [exp (-0.016) = 0.984], 0.054 or [exp (0.054) = 1.055], 0.021 or [exp (0.021) = 1.021], -0.024 or [exp (-0.024) =0.976] and -0.022 or [exp (-0.022) = 0.978], respectively. There was a greater effect of monsoon and TempRain on BI_1 in average. TempRain had a slightly greater effect on BI_1 in Kathmandu than in Chitwan with reference to Lalitpur.

Four significant predictors were included in regression models for *Ae. aegypti* abundance and two for *Ae. albopictus* abundance (Table 6). The partial regression coefficients of significant predictors monsoon, post-monsoon, RelHumidity and C_RelHumidity for *Ae. aegypti* abundance were 5.723 or [exp (5.723) = 305.821], 3.378 or [exp (3.378) = 29.31], -1.271 or [exp (-1.271) = 0.280] and 1.218 or [exp (1.218) = 3.380], respectively. Similarly, the partial regression coefficients of predictors TempRain and RelHumidity for *Ae. albopictus* number were 1.334 or [exp (1.334) = 3.796] and 1.494 or [exp (1.494) = 4.454], respectively. The effect of monsoon was greater than post-monsoon with reference to pre-monsoon on *Ae. aegypti* and RelHumdity had a greater influence on *Ae. albopictus* abundance than TempRain.

**Table 6:** Negative binomial regression model for *Ae. aegypti* and *Ae. albopictus* number

Parameter	*Ae. aegypti*				*Ae. albopictus*			
	Model 23		Model 24		Model 25		Model 26	
	b (SE)	*P* ^a^	b (SE)	*P* ^a^	b (SE)	*P* ^a^	b (SE)	*P* ^a^
(Intercept)	-2.241 (1.302)	0.085	-2.499 (1.171)	0.033	-3.864 (1.602)	0.016	-0.447 (0.277)	0.107
Chitwan	-1.863 (0.929)	0.045			0.571 (0.918)	0.534		
Kathmandu	-0.982 (0.623)	0.115						
Monsoon	3.781 (2.857)	0.186	5.723 (1.586)	<0.0001	6.852 (3.432)	0.046		
Post-monsoon	6.25 (2.903)	0.031	3.378 (1.611)	0.036	1.125 (1.596)	0.481		
TempRain	2.158 (2.186)	0.323			-0.513 (1.839)	0.780	1.334 (0.212)	<0.0001
RelHumidity	-2.008 (0.915)	0.028	-1.271 (0.722)	0.078	-0.447 (0.781)	0.567	1.494 (0.352)	<0.0001
C_TempRain	0.036 (0.635)	0.954			-0.654 (0.663)	0.324		
C_RelHumidity	2.551 (1.160)	0.028	1.218 (0.633)	0.054	0.763 (1.060)	0.472		
K_TempRain	-0.072 (0.535)	0.893			-0.854 (0.699)	0.222		
K_RelHumidity	1.118 (0.698)	0.109			1.055 (0.819)	0.198		

*Abbreviation*: *SE* standard error

^a^Wald Chi-square test

## Discussion

The seasonal prevalence of dengue vectors with respect to their breeding habitats and the influence of meteorological variables on vector indices and population abundance were considered in this study. We found that potential breeding containers as well as containers positive for *Aedes* vectors were greater in number during monsoon season.

Consistent with earlier studies, our findings revealed that discarded tires were highest in number among the positive containers identified for *Aedes* spp. larval breeding [29]. An increase in vehicle numbers due to rapid expansion of urban areas in Nepal has augmented the number of vehicle workshops and tire recycling centers. The reckless dumping of tires in urban areas poses a major problem since they tend to be an ideal breeding habitat for *Aedes* spp. larvae. Discarded tires were also found to be an efficient breeding habitat for *Aedes* spp. by other studies [26, 30–32]. Tires are more likely to retain collected water since they are less likely to be disturbed by human activities. In addition, *Aedes* spp. larvae were also detected in containers made of metal and plastic. Other studies have shown that these types of containers are common breeding habitats [26, 33] and some have noted plastic containers as the most productive breeding habitat [33, 34].

Despite the fact that discarded tires were abundant in terms of the number of breeding containers, their BPR value was not the highest in this study, contrary to the findings of an earlier study in Chitwan District that demonstrated the highest BPR value for discarded tires [29]. Glass container in Kathmandu had the highest BPR value. However, this is due to the fact that the sole water-holding glass container identified was infested with *Aedes* larvae. Similarly, the ratio of metal containers positive for *Aedes* larvae to the total number of water-holding metal containers was higher compared to that of discarded tires in Lalitpur. This made the BPR value of metal containers higher than that of discarded tires, despite a greater number of discarded tires being identified. Similarly, BPR value of metal drum container was highest in Chitwan. These results show that metal and glass containers are suitable breeding habitats for *Aedes* spp. and therefore, these containers should be monitored with equal priority during vector surveillance.

We also found that monsoon season showed the highest values for HI, CI, BI and vector abundance. In the studies conducted in Indonesia, Vietnam, South Korea and Myanmar, higher HI, BI and vector population were found during the rainy season rather than dry season [35–38]. HI, CI and BI are the commonly used larval indices in determining the general distribution and principal habitats of *Aedes* vectors, with HI and BI being the most widely used indices in the monitoring of dengue vector populations [39]. Although these indices have direct relevance to the dynamics of disease transmission, the threshold levels of vector infestation that trigger dengue transmission is influenced by several factors, such as mosquito longevity and immunological status of the human population [26]. Three different risks of HI, with < 0.1% considered as low, 0.1–5% as medium and > 5% as high, were proposed by the Pan American Health Organization to prevent dengue transmission [40, 41]. Our study shows that HI values in all the three surveyed locations fall under high risk and medium risk during monsoon and post-monsoon, respectively. The nil value of HI in dengue endemic Chitwan during pre-monsoon indicates low risk but these values in non-endemic Kathmandu and Lalitpur indicate medium risk. Thus, HI cannot be a reliable predictor of dengue. Although there is no universal critical threshold, an arbitrary threshold of BI = 5 has been applied and the prediction accuracy of dengue epidemic at BI > 5 was 77% [40]. Dengue transmission has been observed with a vector density below the threshold in another study [25]. The BI values in our study were less than 5%. As suggested by other researchers, BI cannot, however, be used as a sole predictor of dengue epidemics [3, 40] although Tun-Lin et al. [42] demonstrated that BI can be considered as the best indicator compared to other indices.

The fact that the greatest numbers of larvae were found during monsoon in this study is linked to the low variation in the temperature range during this season. As shown by the meteorological data for Nepal, the variation in the temperature range (indicated by the standard deviation in Table 1) and difference between maximum and minimum temperature is lower during monsoon compared to other seasons, which can be linked to finding a greater number of *Aedes* spp. larvae during monsoon in this study (Additional file 2: Figure S1). A greater difference between daily maximum and minimum temperature has been attributed to reduced larval survival and increased development time [43]. A previous study in Nepal by Dhimal et al. [14] showed a greater number of adult *Ae. aegypti* and *Ae. albopictus* towards the end of monsoon and post-monsoon season.

Although the adult female population has been shown to peak in November [11], our study found the maximum number of larvae during monsoon. The reason could be that an increase in the number of water-holding containers during monsoon season created sufficient breeding habitats for *Aedes* larvae, resulting in the vector larval stages being dominant. Subsequently, a decline in the number of water-holding containers in post-monsoon season reduced the population of larvae. However, the peak population of adults as observed by Dhimal et al. [11] could be due to the emerged larvae thriving during post-monsoon.

According to a study conducted in Indonesia [38], fewer mosquito larvae were found in the dry season compared to the wet season. Less rainfall means a reduced amount of water retained in containers which affects mosquito breeding. Similarly, the population of *Ae. aegypti* observed by another study was found to be the highest during the period of high rainfall [44]. However, heavy and continuous rainfall has been linked to a reduction in adult mosquito population due to the washing away of immature stages of mosquitoes [45, 46]. Another study found that moderate rain with ideal temperature enhances the *Ae. aegypti* population [33]. *Aedes* abundance has been regulated by temperature rather than by precipitation [47, 48].

In this study, we found the mean *Ae. aegypti* population abundance to be higher in the lowland than in the upland regions while the mean *Ae. albopictus* population abundance was almost identical in both ecological regions (Fig. 3). Chitwan, representing lowland region in this study, is considered a dengue endemic region of Nepal. A higher number of *Ae. aegypti* in the lowland region implies that the abundance of this primary vector is an indicator of risk for dengue transmission; *Ae. aegypti* is known to be the most effective vector for dengue viruses [49]. Similarly, Wijayanti et al. [38] considered a high level of adult *Ae. aegypti* in endemic and sporadic areas as a potential indicator of dengue virus transmission risk in Indonesia. Larval density has been shown to be related to sporadic dengue and its outbreaks in Guangzhou, China [50].

Overall, we found a higher population abundance of *Ae. aegypti* than *Ae. albopictus* in this study (Additional file 2: Figure S1). Since the entomological survey was carried out in urban areas with only man-made containers identified, we were more likely to find a greater number of *Ae. aegypti* since they prefer urban areas [38] while *Ae. albopictus* favor sub-urban areas and natural containers [38, 51]. During this study, we found larvae of both *Ae. aegypti* and *Ae. albopictus* breeding in the same habitat in some of the containers. It has been reported that coexistence of both vectors is possible when a warmer and drier climate alleviates competition from *Ae. albopictus* [52]. Thus, a rise in temperature can facilitate the expansion of *Ae. aegypti* populations.

Meteorological variables are considered some of the environmental factors for increased risk of dengue since they influence the viral replication and vector dynamics. For example, higher temperature has been associated with accelerated virus replication and its reduced extrinsic incubation period in the vector [21, 53]. In addition, elevated temperatures increase mosquito development and host biting rates [18, 54]. However, the increased temperature should be within the optimum range and exceeding this range will result in decreased abundance and reduced survival probabilities [55].

The strength of the relation of meteorological variables with vector indices may be inferred from correlation analysis, but their association is elucidated only by regression analysis. The models that include maximum numbers of significant predictors in our regression analysis were selected for interpretation. The overall effect of the meteorological factor TempRain was found to be significant on vector indices HI_1 and BI_1 compared to relative humidity in the regression analysis. The higher significant effect of temperature and rainfall in the upland region was obvious from the analysis. The temperature in upland is lower compared to lowland. A lower effect of temperature fluctuation on mortality rate of *Ae. aegypti* and *Ae. albopictus* had been demonstrated within the temperature range of 20–30 °C [56]. This temperature range is closer to the temperature records in lowland Chitwan rather than in the upland Kathmandu Valley. Thus, a change in temperature in the uplands will have a greater influence on the mortality rate than in the lowland. Rainfall can potentially make abundant water-holding containers available, resulting in more houses with vector-infested containers. This can be attributed to the increased number of vectors in the monsoon season which showed a significantly higher effect on HI_1 and BI_1 compared to other seasons. However, an increase in the amount of rainfall may not always be favorable for vector abundance [45, 46]. A longer period of moderate rain is important to facilitate the availability of vector breeding sites.

Considering location as predictor variable, Lalitpur had a greater effect on HI_1 compared to Kathmandu and Chitwan. Despite sharing an identical climate pattern, the discrepancy in the effect of Kathmandu and Lalitpur on this response variable can be linked to the effect of non-meteorological factors, which is a limitation of this study. CI showed the weakest relationship of all the entomological indices since it indicated only the proportion of infested containers and not per house or per area [23]. Unlike the number of houses, the number of containers within a locality will not be same throughout the year. Furthermore, some of the containers may be located temporarily, thus, CI cannot reflect the true figure of entomological index in relation to variation of climate in different seasons. We observed that BI had more influence in Chitwan compared to Kathmandu. In the light of Chitwan being an endemic region for dengue transmission, BI should be considered to study the relation of vector indices and dengue incidence.

In terms of location, the effect of relative humidity was greater in Chitwan than Kathmandu. Relative humidity affects the evaporation of water with a higher rate at hotter temperatures [57]. This implies that the influence of relative humidity on vector indices and number is likely to be higher in warmer Chitwan. Relative humidity is one of the crucial factors in the life-cycle of mosquitoes [58]. Higher levels of moisture stimulate the survival of *Aedes* larvae and induce eclosion [59]. Although temperature and rainfall have been frequently addressed factors for vector abundance and dengue transmission, researchers have also considered relative humidity as a contributing factor [17, 60]. The influence of relative humidity on vector indices was greater than temperature-rainfall in the lowland region. The geographical location, the large Narayani River and the proximity of dense tropical forest in lowland Chitwan is likely to contribute to the rise in humidity.

The data on vector indices do not reflect the population count of vector since house or container infested with even a single vector is considered as positive. Hence, we analyzed the effect of predictor variables on *Ae. aegypti* and *Ae. albopictus* abundance and found that the influences are not identical. Other researchers have found that the effects of temperature and relative humidity were not same for *Ae. aegypti* and *Ae. albopictus* [52, 61]. The effect of RelHumidity was higher on *Ae. albopictus. Aedes aegypti* benefitted with higher survival as it is less influenced by humidity compared to *Ae. albopictus* [57].

Any change in climate resulting in the range of meteorological variables conducive for vector breeding is expected to trigger an increase in vector population and thereby affect dengue transmission. However, the effect of climatic factors on dengue transmission and vector distribution is not consistent throughout the world [17, 18, 62, 63]. Ecological and human factors are essential in driving vector-borne diseases [64]. Socio-economic condition, population, water storage behaviors and strength of vector surveillance are also essential considerations for vector prevalence. Rapid urbanization, an increase in international trade and frequent travel have been involved in the spread of vectors and, therefore, increased the global risk of dengue transmission [17, 65, 66]. Thus, vector control is crucial for the containment of dengue transmission. Intending a timely response to dengue outbreaks, the Government of Nepal has adopted a vector control programme which focuses on the search and destruction of *Aedes* spp. larvae during an outbreak. A few locals of Chitwan have volunteered in vector control at the time of outbreaks. In the light of the fact that dengue cases reach its peak during the month of October in Nepal, the vector control programmes are implemented during the post-monsoon season. The prevalence of *Aedes* larvae in monsoon and the more significant effect of monsoon season on HI_1, CI_1, BI_1 and *Ae. aegypti* abundance revealed in this study suggest that the existing practice of vector control during the time of outbreaks may not be sufficient for the effective management of vectors. Executing intensive vector control programme from the onset of a monsoon season with the involvement of a community is fundamental to thwarting off the potential dengue epidemics in the country.

## Conclusions

Our study determined that *Aedes* larval populations peak during the monsoon season when water-holding containers are abundant. Temperature and rainfall contribute to vector indices in upland hilly region whereas relative humidity in lowland plains. The effect of meteorological variables on vector abundance can differ among geographical locations with diverse climate conditions. However, vector dynamics and dengue transmission is complex since other contributing factors such as socio-economic, travel, trade, urbanization and public health intervention are involved. Intense vector surveillance and control measures need to be implemented in lowland Terai as well as the upland hilly regions of Nepal to prevent epidemics of dengue.

## Additional files

Additional file 1: **Table S1.** Number of water-holding containers infested with *Aedes* larvae in the three different locations of the study area in different seasons. (DOCX 16 kb)

Additional file 2: **Figure S1.** Number of *Aedes* species collected in post-monsoon, monsoon and pre-monsoon seasons. (TIF 4144 kb)

Additional file 3: **Table S2.** Principal components analysis for factor extraction (rotation component matrix). (DOCX 11 kb)

Additional file 4: **Table S3.** A goodness-of-fit test for a response variable using generalized linear model (GLM) regression of HI_1. Table S4. A goodness-of-fit test for a response variable using generalized linear model (GLM) regression of CI_1. Table S5. A goodness-of-fit test for a response variable using generalized linear model (GLM) regression of BI_1. Table S6. A goodness-of-fit test for a response variable using generalized linear model (GLM) regression of *Ae. aegypti* numbers and *Ae. albopictus* numbers. (DOCX 21 kb)

## Abbreviations

BI: Breteau index
BPR: Breeding preferences ratio
CI: Container index
DENV: Dengue virus
GLM: Generalized linear model
GLMM: Generalized linear mixed model
HI: House index
PCA: Principal components analysis
