# Supplementary material for: Effect of meteorological factors on the seasonal prevalence of dengue vectors in upland hilly and lowland Terai regions of Nepal
Source: Parasit Vectors. 2019 Jan 18;12:42. doi: 10.1186/s13071-019-3304-3 (PMC6339416; doi:10.1186/s13071-019-3304-3)
Supplement: Supplementary file 1 — Table S1. Number of water-holding containers infested with Aedes larvae in the three different locations of the study area in different seasons. (DOCX 16 kb) [file 13071_2019_3304_MOESM1_ESM.docx]

**Additional file 1: Table S1.** Number of water-holding containers infested with *Aedes* larvae in the three different locations of the study area in different seasons

| **Type of container** | **Location** | **Water-holding containers observed**  **_____________________________**  **Monsoon Post-monsoon Pre-monsoon** | | | **Total observed** | **Containers positive for *Aedes* larvae**  **________________________________**  **Monsoon Post-monsoon Pre-monsoon** | | | **Total positive container** | **Percentage of positive container (%)** | **Breeding Preference Ratio (BPR)** |
| --- | --- | --- | --- | --- | --- | --- | --- | --- | --- | --- | --- |
| Discarded tire | Kathmandu | 222 | 22 | 37 | 281 | 53 | 5 | 2 | 60 | 21.35 | 1.05 |
|  | Lalitpur | 178 | 26 | 43 | 247 | 82 | 4 | 6 | 92 | 37.24 | 1.03 |
|  | Chitwan | 135 | 15 | 5 | 155 | 44 | 2 | 0 | 46 | 29.67 | 0.94 |
| Glass | Kathmandu | 1 | 0 | 0 | 1 | 1 | 0 | 0 | 1 | 100 | 4.9 |
|  | Lalitpur | 0 | 0 | 0 | 0 | 0 | 0 | 0 | 0 | 0 | 0 |
|  | Chitwan | 11 | 0 | 0 | 11 | 3 | 0 | 0 | 3 | 27.27 | 0.86 |
| Metal container | Kathmandu | 8 | 2 | 0 | 10 | 2 | 0 | 0 | 2 | 20 | 0.99 |
|  | Lalitpur | 7 | 0 | 1 | 8 | 4 | 0 | 0 | 4 | 50 | 1.43 |
|  | Chitwan | 2 | 0 | 0 | 2 | 2 | 0 | 0 | 2 | 100 | 3.08 |
| Metal drum | Kathmandu | 5 | 0 | 1 | 6 | 1 | 0 | 0 | 1 | 16.6 | 0.83 |
|  | Lalitpur | 6 | 0 | 1 | 7 | 3 | 0 | 0 | 3 | 42.8 | 1.2 |
|  | Chitwan | 0 | 1 | 0 | 1 | 0 | 1 | 0 | 1 | 100 | 3.16 |
| Plastic bucket | Kathmandu | 4 | 0 | 0 | 4 | 1 | 0 | 0 | 1 | 25 | 1.24 |
|  | Lalitpur | 1 | 0 | 0 | 1 | 0 | 0 | 0 | 0 | 0 | 0 |
|  | Chitwan | 2 | 0 | 0 | 2 | 1 | 0 | 0 | 1 | 50 | 1.58 |
| Plastic container | Kathmandu | 4 | 3 | 0 | 7 | 0 | 1 | 0 | 1 | 14.28 | 0.7 |
|  | Lalitpur | 2 | 0 | 0 | 2 | 0 | 0 | 0 | 0 | 0 | 0 |
|  | Chitwan | 1 | 0 | 0 | 1 | 0 | 0 | 0 | 0 | 0 | 0 |
| Plastic cup | Kathmandu | 19 | 0 | 0 | 19 | 1 | 0 | 0 | 1 | 5.26 | 0.26 |
|  | Lalitpur | 10 | 0 | 0 | 10 | 1 | 0 | 0 | 1 | 10 | 0.27 |
|  | Chitwan | 0 | 0 | 0 | 0 | 0 | 0 | 0 | 0 | 0 | 0 |
| Plastic drum | Kathmandu | 2 | 0 | 0 | 2 | 0 | 0 | 0 | 0 | 0 | 0 |
|  | Lalitpur | 2 | 0 | 0 | 2 | 0 | 0 | 0 | 0 | 0 | 0 |
|  | Chitwan | 1 | 0 | 0 | 1 | 0 | 0 | 0 | 0 | 0 | 0 |
| Porcelain cup | Kathmandu | 1 | 0 | 0 | 1 | 0 | 0 | 0 | 0 | 0 | 0 |
|  | Lalitpur | 0 | 0 | 0 | 0 | 0 | 0 | 0 | 0 | 0 | 0 |
|  | Chitwan | 0 | 0 | 0 | 0 | 0 | 0 | 0 | 0 | 0 | 0 |
| Styrofoam | Kathmandu | 1 | 0 | 0 | 1 | 0 | 0 | 0 | 0 | 0 | 0 |
|  | Lalitpur | 0 | 0 | 0 | 0 | 0 | 0 | 0 | 0 | 0 | 0 |
|  | Chitwan | 0 | 0 | 0 | 0 | 0 | 0 | 0 | 0 | 0 | 0 |
|  |  | **625** | **69** | **88** | **782** | **199** | **13** | **8** | **220** | **28.13** |  |
