# Supplementary material for: Effect of meteorological factors on the seasonal prevalence of dengue vectors in upland hilly and lowland Terai regions of Nepal
Source: Parasit Vectors. 2019 Jan 18;12:42. doi: 10.1186/s13071-019-3304-3 (PMC6339416; doi:10.1186/s13071-019-3304-3)
Supplement: Supplementary file 3 — Table S2. Principal components analysis for factor extraction (rotation component matrix). (DOCX 11 kb) [file 13071_2019_3304_MOESM3_ESM.docx]

**Additional file 3: Table S2.** Principal components analysis for factor extraction (Rotation component Matrix)

| Location |  | Components | |
| --- | --- | --- | --- |
|  |  | Factor 1(TempRain) | Factor 2 (Relhumidity) |
| Kathmandu | Maximum temperature | 0.970 | -0.74 |
|  | Minimum temperature | 0.983 | -0.018 |
|  | Rainfall | 0.977 | 0.182 |
|  | Relative humidity | 0.023 | 0.999 |
|  |  | Factor 1(TempRain) | Factor 2 (Relhumidity) |
| Lalitpur | Maximum temperature | 0.991 | 0.054 |
|  | Minimum temperature | 0.959 | 0.272 |
|  | Rainfall | 0.943 | 0.309 |
|  | Relative humidity | 0.189 | 0.981 |
|  |  | Factor 1(TempRain) | Factor 2 (Relhumidity) |
| Chitwan | Maximum temperature | 0.968 | -0.163 |
|  | Minimum temperature | 0.990 | -0.071 |
|  | Rainfall | 0.811 | 0.527 |
|  | Relative humidity | -0.086 | 0.982 |

Extraction method: Principal Component Analysis.

Rotation method: Varimax with Kaiser normalization
