# Supplementary material for: Effect of meteorological factors on the seasonal prevalence of dengue vectors in upland hilly and lowland Terai regions of Nepal
Source: Parasit Vectors. 2019 Jan 18;12:42. doi: 10.1186/s13071-019-3304-3 (PMC6339416; doi:10.1186/s13071-019-3304-3)
Supplement: Supplementary file 4 — Table S3. A goodness-of-fit test for a response variable using generalized linear model (GLM) regression of HI_1. Table S4. A goodness-of-fit test for a response variable using generalized linear model (GLM) regression of CI_1. Table S5. A goodness-of-fit test for a response variable using generalized linear model (GLM) regression of BI_1. Table S6. A goodness-of-fit test for a response variable using generalized linear model (GLM) regression of Ae. aegypti numbers and Ae. albopictus numbers. (DOCX 21 kb) [file 13071_2019_3304_MOESM4_ESM.docx]

**Additional file 4: Table S3.** A goodness of fit test for a response variable using Generalized Linear Model (GLM) regression of HI_1

| **Models Link function** | **No. of predictor** | | **Deviance** | | | | **Omnibus Test** | | **AIC** | **BIC** |
| --- | --- | --- | --- | --- | --- | --- | --- | --- | --- | --- |
|  | S* | NS* | -2LL | df | (-2LL/df) | Dispersion parameter | Likelihood ratio  χ^2^ (df) | Sig |  |  |
| 1: Normal: Identity | 1 | 9 | 2000.95 | 76 | 26.33 | 22.99 | 128.21(10) | 0.00 | 543.68 | 573.27 |
| 2: Normal: Log | 1 | 9 | 2014.41 | 76 | 26.51 | 23.15 | 127.63(10) | 0.00 | 544.27 | 573.86 |
| 3: Gamma: Identity | 2 | 8 | 56.03 | 76 | 00.74 | 00.59 | 99.50(10) | 0.00 | 475.87 | 505.87 |
| 4: Gamma: Log | 5 | 5 | 57.53 | 76 | 00.76 | 00.60 | 96.97(10) | 0.00 | 478.39 | 507.98 |
| 5: Tweedle (1.5): Identity | 2 | 8 | 109.27 | 76 | 01.44 | 01.07 | 140.95(10) | 0.00 | 484.90 | 510.28 |
| 6: Tweedle (1.5): Log | 4 | 6 | 111.60 | 76 | 01.47 | 01.09 | 138.79(10) | 0.00 | 487.06 | 512.44 |
| 7: Gamma: Log | 5 | 0 | 60.57 | 81 | 00.75 | 00.63 | 92.04(5) | 0.00 | 473.32 | 490.58 |
| 8: Gamma: Log | 4 | 0 | 74.22 | 82 | 00.95 | 00.76 | 72.40(4) | 0.00 | 490.96 | 505.76 |
| 9: Gamma: Log | 4 | 0 | 78.01 | 82 | 0.951 | 00.79 | 67.54(4) | 0.00 | 495.83 | 510.62 |

* S refers to significance predictors and NS refers to not-significant predictors at most 10% level of significance.

**Additional file 4: Table S4.** A goodness of fit test for a response variable using Generalized Linear Model (GLM) regression of CI_1

| **Models Link function** | **No. of predictor** | | **Deviance** | | | | **Omnibus Test** | | **AIC** | **BIC** |
| --- | --- | --- | --- | --- | --- | --- | --- | --- | --- | --- |
|  | S* | NS* | -2LL | df | (-2LL/df) | Dispersion parameter | Likelihood ratio  χ^2^ (df) | Sig |  |  |
| 10: Normal: Identity | 2 | 8 | 18738.48 | 76 | 246.56 | 215.39 | 54.09(10) | 0.00 | 742.51 | 767.89 |
| 11: Normal: Log | 4 | 6 | 17812.27 | 76 | 234.37 | 204.74 | 58.50(10) | 0.00 | 738.10 | 763.48 |
| 12: Gamma: Log | 4 | 6 | 128.70 | 76 | 1.69 | 1.25 | 50.63(10) | 0.00 | 621.18 | 646.55 |
| 13: Tweedle (1.5): Identity | 3 | 7 | 349.54 | 76 | 4.60 | 2.57 | 80.71(10) | 0.00 | 664.88 | 689.47 |
| 14: Tweedle (1.5): Log | 6 | 4 | 355.34 | 76 | 4.68 | 2.60 | 78.47(10) | 0.00 | 666.33 | 691.71 |
| 15: Gamma: Log | 5 | 0 | 132.96 | 81 | 1.64 | 1.28 | 47.25(5) | 0.00 | 611.76 | 627.60 |

* S refers to significance predictors and NS refers to not-significant predictors at most 10% level of significance.

**Additional file 4: Table S5.** A goodness of fit test for a response variable using Generalized Linear Model (GLM) regression of BI_1

| **Models Link function** | **No. of predictor** | | **Deviance** | | | | **Omnibus Test** | | **AIC** | **BIC** |
| --- | --- | --- | --- | --- | --- | --- | --- | --- | --- | --- |
|  | S* | NS* | -2LL | df | (-2LL/df) | Dispersion parameter | Likelihood ratio  χ^2^ (df) | Sig |  |  |
| 16: Normal: Identity | 3 | 7 | 0.103 | 76 | 0.001 | 0.001 | 59.24(10) | 0.00 | -311.17 | -285.79 |
| 17: Normal: Log | 4 | 6 | 0.103 | 76 | 0.001 | 0.001 | 59.61(10) | 0.00 | -315.76 | -286.17 |
| 18: Gamma: Identity | 5 | 5 | 0.081 | 76 | 0.001 | 0.001 | 66.21(10) | 0.00 | -327.78 | -302.19 |
| 19: Gamma: Log | 4 | 6 | 0.081 | 76 | 0.001 | 0.001 | 66.55(10) | 0.00 | -327.90 | -302.53 |
| 20: Tweedle (1.5): Identity | 3 | 7 | 0.086 | 76 | 0.001 | 0.001 | 64.48(10) | 0.00 | -327.81 | -298.22 |
| 21: Tweedle (1.5): Log | 4 | 6 | 0.086 | 76 | 0.001 | 0.001 | 64.83(10) | 0.00 | -323.94 | -298.57 |
| 22: Gamma: Log | 6 | 0 | 0.082 | 80 | 0.001 | 0.0013 | 64.90(6) | 0.00 | -338.46 | -318.74 |

* S refers to significance predictors and NS refers to not-significant predictors at most 10% level of significance.

**Additional file 4: Table S6.** A goodness of fit test for a response variable using Generalized Linear Model (GLM) regression of *Ae. aegypti* number and *Ae. Albopictus* number

| **Models** | **Link function** | **No. of Predictor** | | **Deviance** | | | | | **Omnibus Test** | | | **AIC** | **BIC** |
| --- | --- | --- | --- | --- | --- | --- | --- | --- | --- | --- | --- | --- | --- |
|  |  | S* | NS* | -2LL | df | (-2LL/df) | | Dispersion  parameter | | Likelihood Ratio  χ^2^ (df) | Sig |  |  |
| 23: Negative Binomial: Log | | 4 | 6 | 70.22 | 75 | 0.936 | 2.266 | | 52.51(10) | | .000 | 354.96 | 380.34 |
| 24: Negative Binomial: Log | | 4 | 4 | 70.22 | 81 | 0.867 | 2.860 | | 42.38(4) | | .000 | 349.88 | 363.67 |
| 25: Negative Binomial: Log | | 1 | 8 | 60.81 | 76 | 0.800 | 1.346 | | 58.73(9) | | .000 | 257.78 | 281.38 |
| 26: Negative Binomial: Log | | 2 | 0 | 62.93 | 83 | 0.758 | 2.025 | | 44.14(2) | | .000 | 255.34 | 264.72 |

Note: *S refers to significant predictors and NS for not-significant predictors at most 10% level of significance.
